# The Price She Pays

**Published:** 1913-07

**Authors:** W. C. Poole


### The Price She Pays.

BY W. C. POOLE.

She sins her sin but not alone,
Another sinned with her that day,
And yet for ages men have known
She must the full price pay;—
She bears the curse of sin while he
To blight another life goes free.

She sins her sin of scarlet hue,
She bears its guilt and shame,
She who was once like morning dew,
Can never right her name;
And he goes forth to call her fool
Who just to please him was his tool.

O God make bare thy mighty arm,
Let Justice reign one day,
And smite the man who dares to harm,
A woman on life's way;
And in the hearts of men awake
A sense of Justice for her sake.

And if I wrongly ask again,
Lord God, I pray forgive,
But mark the man as thou didst Cain
Unfit with men to live;
Give him a mark that all may know
The jungle beast where he may go.

—*The Light.*

---

INGRATITUDE.—Medical Man: Jobson has done the meanest thing ever I heard of. He came to my house the other night, ate a big dinner, got indigestion, and then went to another doctor to be cured.—*Tit-Bits.*

---

THE INEVITABLE.—"My husband is particularly liable to sea sickness, captain," remarked a lady passenger. "Could you tell him what to do in case of an attack?" "'Taint necessary, ma'am," replied the captain. "He'll do it."—*Tit-Bits.*

